# Characteristics of compounds that cross the blood-brain barrier

**DOI:** 10.1186/1471-2377-9-S1-S3

**Published:** 2009-06-12

**Authors:** William A Banks

**Affiliations:** 1 Veterans Affairs Medical Center-St Louis, 915 N. Grand Blvd, St. Louis, MO 63106, USA

## Abstract

Substances cross the blood-brain barrier (BBB) by a variety of mechanisms. These include transmembrane diffusion, saturable transporters, adsorptive endocytosis, and the extracellular pathways. Here, we focus on the chief characteristics of two mechanisms especially important in drug delivery: transmembrane diffusion and transporters. Transmembrane diffusion is non-saturable and depends, on first analysis, on the physicochemical characteristics of the substance. However, brain-to-blood efflux systems, enzymatic activity, plasma protein binding, and cerebral blood flow can greatly alter the amount of the substance crossing the BBB. Transport systems increase uptake of ligands by roughly 10-fold and are modified by physiological events and disease states. Most drugs in clinical use to date are small, lipid soluble molecules that cross the BBB by transmembrane diffusion. However, many drug delivery strategies in development target peptides, regulatory proteins, oligonucleotides, glycoproteins, and enzymes for which transporters have been described in recent years. We discuss two examples of drug delivery for newly discovered transporters: that for phosphorothioate oligonucleotides and for enzymes.

## Introduction

**Figure 1:**
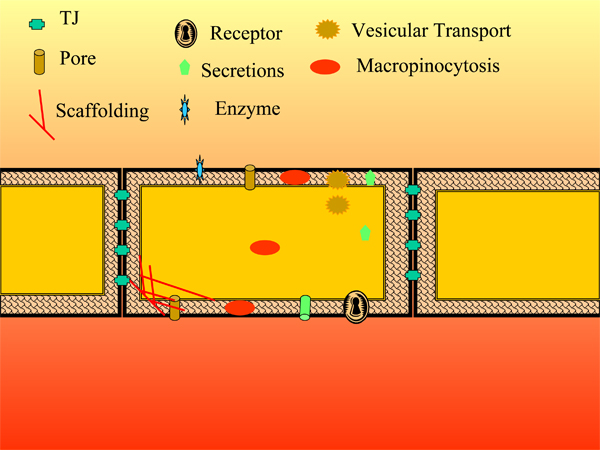
**A generic brain barrier**. Adult mammalian brain barriers reduce uncontrolled leakage by constituting a monolayer of cells characterized by intercellular tight junctions, decreased macropinocytosis, and decreased fenetrae. Variations on this theme are seen at the vascular brain barrier, blood-CSF barrier, and the specialty CNS barriers such as the blood-retinal barrier. Most brain barriers have a combination of the other features shown. Pores are saturable transporters that can be energy dependent (as exemplified by P-glycoprotein) or energy independent (GLUT-1), located at the luminal or abluminal membrane, and transport bidirectionally or unidirectionally into or out of the cytoplasm. Saturable transport can also be vesicular based and brain barriers likely have many types of vesicular systems (for example, receptor-mediated transcytosis, clathrin-dependent transport, podocytosis, and caveolae). Scaffolding (for example, actin) is likely highly dynamic and involved in tight junction function and vesicular trafficking. Barrier cells contain receptors (binding sites coupled to intracellular machinery) as well as transporters (binding sites coupled to machinery involved in translocation of the ligand). Brain barriers are enzymatically active and this activity can act as another layer of barrier, and they can secrete substances such as cytokines, nitric oxide, and prostaglandins from either their CNS or peripheral side.

The blood-brain barrier (BBB) represents a major obstacle to the delivery of drugs to the central nervous system (CNS). The BBB consists of several barriers in parallel, with the two that are best described being the vascular BBB, consisting primarily of the capillary bed, and the blood-cerebrospinal fluid (blood-CSF) barrier, consisting primarily of the choroid plexus [1]. Although drug delivery tends to focus on the vascular BBB, the blood-CSF barrier also presents special opportunities [2]. At both sites, the BBB is formed by a monolayer of cells that are cemented together by tight junctions and have other mechanisms that control or retard leakage of plasma into the CNS (Figure 1). Barrier function at the BBB often depends on more than physical considerations and can be bolstered by enzymatic and brain-to-blood transporter functions [3].

The BBB serves roles other than that of blocking circulating substances from entering the CNS. It also facilitates and regulates the entry of many substances that are critical to CNS function and secretes substances into the blood and CNS. These extra-barrier functions allow the BBB to influence the homeostatic, nutritive, and immune environments of the CNS and to regulate the exchange of informational molecules between the CNS and blood [4]. An understanding of the barrier and extra-barrier aspects of BBB physiology is critical to developing drugs that can access the CNS [5].

## General characteristics of the blood-brain barrier

Three major modifications to the capillary bed of the brain prevent the formation of a plasma ultrafiltrate in the CNS (Figure 1): tight junctions that cement together brain endothelial cells that are in apposition, a greatly reduced rate of pinocytosis, and a lack of intracellular fenestrations [6]. These modifications prevent the unregulated leakage of serum proteins into the CNS under normal conditions. Substances are still able to cross the vascular BBB by a variety of mechanisms. These mechanisms include transmembrane diffusion, saturable transport, adsorptive endocytosis, and the extracellular pathways. Below, we discuss transmembrane diffusion and saturable transporters. Reviews on adsorptive endocytosis and the extracellular pathways can be found elsewhere [7,8].

### Transmembrane diffusion

Most drugs cross the BBB by transmembrane diffusion [9]. This is a non-saturable mechanism that depends on the drug melding into the cell membrane. A low molecular weight and high degree of lipid solubility favor crossing by this mechanism. However, a drug taken up by the membranes that form the BBB must then partition into the aqueous environment of the brain's interstitial fluid to exert an effect. As a result, a substance that is too lipid soluble can be sequestered by the capillary bed and not reach the cells behind the BBB. Lipid solubility also favors uptake by the peripheral tissues; this, in turn, lowers the concentration of the drug in blood. Thus, while lipid solubility can increase transport rate across the BBB, it can also lower the amount of the drug presented to the BBB. The percent of administered drug entering the brain is determined by both the rate of transport across the BBB and the amount of drug presented to the brain [5]. Use of lipid solubility to improve drug delivery to the brain must thus find the balance between increased permeation of the BBB and decreased concentrations in blood.

Factors in addition to lipid solubility affect the ability of a drug to partition from blood into the BBB. These include charge, tertiary structure and degree of protein binding. Chief among these secondary factors, however, is molecular weight. The best approximation of the influence of size on BBB penetration is that it is inversely related to the square route of molecular weight. Reviews often quote an absolute cut-off of 400 to 600 Da for penetration of the BBB, but these arise from a misreading of the literature. The 'Rule of 5' of Lipinski found that from a library of drugs selected for gastrointestinal absorption, few substances were over 500 Da [10]. Some reviewers have uncritically applied the Rule of 5, including this one, to the BBB. A study of 27 substances by Levin [11] found that the four drugs in this groups with molecular weights over 400 Da had no measurable brain uptake. However, it is now known that these substances are all substrates for P-glycoprotein, a major brain-to-blood, or efflux, pump located at the BBB that prevents or greatly retards a large number of small, lipid soluble molecules from entering the CNS [12,13]. Peptides and proteins with molecular weights in excess of 600 Da are known to cross the BBB in amounts sufficient to affect CNS function. Early examples include delta sleep-inducing peptide and enkephalin analogs. The largest substance found to date to cross the BBB by the mechanism of transmembrane diffusion is cytokine-induced neutrophil chemoattractant-1 (CINC-1) at 7,800 Da [14].

Lipophilic substances of low molecular weight tend to be substrates for P-glycoprotein [12]. Brain-to-blood efflux by P-glycoprotein can greatly limit the rate of uptake by the BBB and is a major obstacle in drug development. The pharmacogenomics of P-glycoprotein show that about 30% of the population overexpress it and so are less sensitive to the CNS effects of its ligands, while about 25% of the population underexpress it [15]. Such individual variation has been linked to sensitivity to drugs for the treatment of AIDS and epilepsy [15,16].

### Saturable transport systems

Some drugs or substances used for drug-like effects cross the BBB by use of saturable transport systems. L-DOPA and caffeine are examples as are vitamins such as B12 and B6 [17]. The uptake rate across the BBB for an endogenous ligand of a transporter is roughly about 10 times higher than would be expected if it crossed by transmembrane diffusion [18]. Additionally, many of the transporters for regulatory molecules, such as peptides and regulatory proteins, are taken up selectively by specific brain regions [19,20]. Thus, exploitation of transporters offer the drug development field not only high uptake rates for large, water soluble compounds but targeting to specific regions of the CNS.

Efflux transporters have the opposite effect to influx transporters in that they decrease the uptake rate of potential drugs [13]. P-glycoprotein has been discussed above, but the BBB possesses many other efflux transporters. As discussed below, peptide transport system-6 (PTS-6) retards the accumulation from blood by brain of the 27 amino acid form of pituitary adenylate cyclase activating polypeptide (PACAP27) [21].

The rate at which saturable systems transport their ligands across the BBB is often regulated. For flow-dependent substances such as glucose, transport rate is a function of cerebral blood flow [5]. For substances that are more slowly transported, a variety of agents have been found to alter transport. For example, leucine regulates the transport rate of peptide transport system-1 (PTS-1) [22] and epinephrine and triglycerides affect leptin, ghrelin, and insulin transport [23-25].

Under physiological conditions, the BBB transporters adapt to serve the needs of the CNS. Uncoupling between BBB functions and CNS needs is accompanied by disease states [26]. For example, decreased leptin transport is associated with peripheral leptin resistance in obesity [27] and decreased efflux of amyloid beta protein is associated with Alzheimer's disease [28].

## General strategies for drug transport

A great deal of current effort towards drug development is directed towards *in silico *analysis and high-throughput screening. Such efforts limit drug discovery to substances crossing the BBB by transmembrane diffusion. They also limit discovery to the main parameters in the library used as the basis of computation. *In silico *methods are likely less efficient in the search for CNS drug candidates than in the search for those absorbed by the gastrointestinal tract because of a number of parameters that can modify or override transmembrane diffusion: cerebral blood flow, influx and efflux transporters, protein binding in the blood, clearance from blood, sequestration by BBB tissues, and enzymatic activity by peripheral tissues, blood, the CNS and at the BBB [5].

Many approaches to drug development have attempted to harness transporters. The usual approach is a version of the 'Trojan horse' strategy [29]. Here, a substance that does not cross the BBB is coupled to a substance that does. Such coupling can have the added benefit of improving peripheral pharmacokinetics. Unfortunately, the resulting hybrid compound is often not recognized by the original transporter or the transporter/hybrid compound is routed to lysosomes for destruction. Hybrids coupled to other substances may use other vesicular pathways across the BBB. Unfortunately, the cell biology of BBB vesicular systems is poorly understood and this impairs exploitation of promising leads.

Development of analogs of transported ligands has been slow. Many endogenous substances that could be the basis of CNS drugs, such as the feeding hormones and cytokines, are transported across the BBB [30]. However, the endogenous compounds have poor peripheral pharmacokinetics and this limits their usefulness [3]. Analogs would have to retain their affinity for both the BBB transporter and for the CNS receptor while becoming less favorable for peripheral enzymes and clearance mechanisms.

When disease states affect the BBB or the BBB is itself impaired, then it becomes a therapeutic target in its own right [26]. A classic example is multiple sclerosis in which the BBB becomes leaky and allows the entry of immune cells into the CNS. However, the passage of immune cells across the BBB is a highly regulated process [31] and the leakage is likely a byproduct of immune cell trafficking and not the other way round [32]. Obviously, the luminal surface of the capillary bed does not require passage across the BBB and, hence, drug strategies used to target peripheral tissues are applicable to this half of the BBB. Luminal receptors that induce brain endothelial cells to secrete into the CNS substances such as prostaglandins, cytokines, and nitric oxide are also readily targetable. This suggests that the BBB itself could be used as the source of CNS 'drugs'.

'Bypassing' the BBB can also be an effective strategy, especially for selected cases or situations. For example, intrathecal administration for delivery of drug to the brain is ineffectual for small, lipid soluble drugs [33]. However, this route may be an option for large regulatory proteins with negligible brain-to-blood efflux [34]. Intranasal delivery of drugs, including peptides [35], shows a great deal of promise [36]. Nasal delivery of insulin, for example, has had positive effects in treating Alzheimer's disease [37,38].

## Examples and special cases

The various strategies used to develop drugs towards the CNS are meeting with varied levels of success. Those that consider the special features of the BBB rather than 'black boxing' it, attempt to understand the underlying mechanisms of promising leads, and consider the peripheral pharmacokinetics of the candidate drug should have advantages. However, there is a great deal that is unknown about the BBB that would be of great use to CNS drug development. For example, there are likely a great many BBB transporters yet to be discovered. Below, we consider two newly discovered transporters and their early applications to drug development.

Antisense molecules have been assumed to be incapable of crossing the BBB. The rapid clearance of any mRNA material in the circulation would certainly justify this assumption. However, enzymatically resistant analogs such as peptide nucleic acids and phosphorothioate oligonucleotides (PONs) can cross the BBB in sufficient amounts to affect CNS function [39,40]. The PONs are transported across the BBB by a saturable transport system. This transporter has been used to deliver an antisense molecule directed against amyloid precursor protein, which effectively reverses the cognitive deficit in an animal model of Alzheimer's disease. PONs have also been directed at the efflux transporter of PACAP27 [41]. The PONs reduce expression of the transporter, increase PACAP27 retention by brain after its peripheral administration, and improve outcomes in animal models of stroke and Alzheimer's disease. These results show that targeting efflux systems at the BBB with antisense molecules can improve drug delivery to the brain.

Mucopolysaccharidoses consist of a number of diseases in which missing enzymes lead to the accumulation of glycosaminoglycans in brain and peripheral tissues. Enzyme replacement clears the glycosaminoglycans from the peripheral tissues, but not from the CNS as the enzymes do not cross the BBB. However, it was recently discovered that the mannose-6 phosphate receptor acts as a saturable transporter at the neonatal BBB [42,43]. As a result, enzyme given to the neonate is effective in clearance of glycosaminoglycans from the CNS [44-46]. Unfortunately, this transport function is lost with development. Recent work has shown that transporter function can be re-induced in the adult with epinephrine [47]. How epinephrine invokes this re-induction of activity is unclear, but it may be a useful strategy for delivery of enzyme to the CNS.

## Conclusion

The BBB is a complex regulatory interface that possesses barrier, secretory, enzymatic, and transporter activities. Transmembrane diffusion, harnessing of transporters, adsorptive endocytosis, and extracellular pathways are some of the mechanisms being exploited for drug delivery. Unfortunately, our understanding of the BBB in many areas, especially those of saturable transport systems and vesicular pathways, is limited. Future successes in CNS drug discovery will likely result from an interplay of exploratory research and rational drug development.

## List of abbreviations used

BBB: blood-brain barrier; CNS: central nervous system; CSF: cerebrospinal fluid; PACAP: pituitary adenylate cyclase activating polypeptide; PON: phosphorothioate oligonucleotide.

## Competing interests

The author is a shareholder in EDUNN, a biotech company investigating the development of antisense molecules for the treatment of Alzheimer's and other CNS diseases.
